# *Anopheles arabiensis* hotspots along intermittent rivers drive malaria dynamics in semi-arid areas of Central Ethiopia

**DOI:** 10.1186/s12936-021-03697-z

**Published:** 2021-03-17

**Authors:** Kasahun Eba, Tibebu Habtewold, Delenasaw Yewhalaw, George K. Christophides, Luc Duchateau

**Affiliations:** 1 Biometrics Research Centre, Faculty of Veterinary Medicine, Ghent University, Merelbeke, Belgium; 2 Department of Environmental Health Science and Technology, Jimma University, P.O. Box 378, Jimma, Ethiopia; 3 Department of Life Sciences, Imperial College London, London, UK; 4 School of Medical Laboratory Sciences, Jimma University, P.O.Box 378, Jimma, Ethiopia; 5 Tropical and Infectious Diseases Research Center, Jimma University, P.O.Box 378, Jimma, Ethiopia

**Keywords:** Clusters, Ethiopia, Intermittent rivers, Malaria, Seasonal dynamics, Transmission intensity

## Abstract

**Background:**

Understanding malaria vector’s population dynamics and their spatial distribution is important to define when and where the largest infection risks occur and implement appropriate control strategies. In this study, the seasonal spatio-temporal dynamics of the malaria vector population and transmission intensity along intermittent rivers in a semi-arid area of central Ethiopia were investigated.

**Methods:**

Mosquitoes were collected monthly from five clusters, 2 close to a river and 3 away from a river, using pyrethrum spray catches from November 2014 to July 2016. Mosquito abundance was analysed by the mixed Poisson regression model. The human blood index and sporozoite rate was compared between seasons by a logistic regression model.

**Results:**

A total of 2784 adult female *Anopheles gambiae *sensu lato (*s.l*.) were collected during the data collection period. All tested mosquitoes (n = 696) were identified as *Anopheles arabiensis* by polymerase chain reaction. The average daily household count was significantly higher (*P* = 0.037) in the clusters close to the river at 5.35 (95% CI 2.41–11.85) compared to the clusters away from the river at 0.033 (95% CI 0.02–0.05). Comparing the effect of vicinity of the river by season, a significant effect of closeness to the river was found during the dry season (*P* = 0.027) and transition from dry to wet season (*P* = 0.032). Overall, *An. arabiensis* had higher bovine blood index (62.8%) as compared to human blood index (23.8%), ovine blood index (9.2%) and canine blood index (0.1%). The overall sporozoite rate was 3.9% and 0% for clusters close to and away from the river, respectively. The overall *Plasmodium falciparum* and *Plasmodium vivax* entomologic inoculation rates for *An. arabiensis* in clusters close to the river were 0.8 and 2.2 infective bites per person/year, respectively.

**Conclusion:**

Mosquito abundance and malaria transmission intensity in clusters close to the river were higher which could be attributed to the riverine breeding sites. Thus, vector control interventions including targeted larval source management should be implemented to reduce the risk of malaria infection in the area.

**Supplementary Information:**

The online version contains supplementary material available at 10.1186/s12936-021-03697-z.

## Background

The global decline of malaria incidence due to scaling up of existing interventions has led to increased interest by many endemic countries to plan for malaria elimination. In such an elimination agenda, the understanding of the spatio-temporal heterogeneity in areas of unstable transmission is of primordial importance [1, 2]. Spatio-temporal heterogeneity leads to restricted areas, so-called malaria hotspots, where mosquito breeding and transmission is ongoing even in the dry season [3] and from where malaria transmission can spread to a wider area when conditions are more conducive [4]. The smallest spatial unit capable of supporting transmission is the household, where peri-domestic transmission occurs [5].

Malaria case distribution is characterized by spatio-temporal heterogeneity in unstable transmission areas [2, 6]. Thus, malaria distribution in low incidence settings appears patchy, and local transmission hotspots are a continuous source of infection [7, 8]. Also in malaria endemic regions micro-epidemiologic variation in malaria incidence and transmission exists and high-risk areas (hotspots) for malaria infection are constantly present [9, 10]. Hence, it is critical to define malaria spatial units at micro-epidemiological level, identify spatial heterogeneity of transmission and assess the effectiveness of malaria control programmes within these units. To this end, studies generated fine scale maps of malaria endemicity and identified spatial units that have higher transmission than their surroundings [11–13].

Though malaria transmission intensity is associated with rainfall apart from other factors, there is evidence that transmission persists late in the dry season. A study in Senegal supports the hypothesis that malaria transmission is maintained during the dry season in an area of low and seasonal transmission [14]. Malaria parasite transmission and clinical disease are characterized by important micro-geographic variations, even between adjacent villages, households or families [15]. Among other factors, the presence or absence of breeding sites contributes to heterogeneity of malaria transmission. A study in Bandiagara, Mali, confirms that the existence of marked spatial heterogeneity of malaria transmission was related to the occurrence of seasonal breeding sites [16].

Understanding the dynamics of malaria transmission and knowing the spatial vector distribution helps to define when and where the largest infection risks occur and supports the development of appropriate and targeted control strategies [17]. Moreover, it is important when malaria transmission rate is on decline, that the control measures are directed towards the transmission foci [18].

Patterns of malaria transmission along river basins was studied in Eastern Sudan in which infectivity and transmission rates increased with proximity to the river following peak rainfall and subsequent recession of rivers [19]. In Ethiopia, malaria transmission is seasonal, and is determined by altitude, rainfall and temperature in addition to other local factors [20]. Despite its seasonality, there are substantial small scale spatial variations in malaria transmission [21]. This is supported by a study that showed a high variation in malaria incidence and *Anopheles* mosquito abundance and distribution among villages [22].

However, currently, there is only limited evidence that local hotspots fuel transmission and little is known about the seasonal variation and intensity of malaria transmission in clusters close to intermittent rivers in Ethiopia. Such information is required for planning and implementing effective vector control interventions. Therefore, this study aimed at investigating seasonal dynamics of malaria vectors and transmission intensity along intermittent rivers in a semi-arid area of central Ethiopia.

## Methods

### Study area

**Fig. 1:**
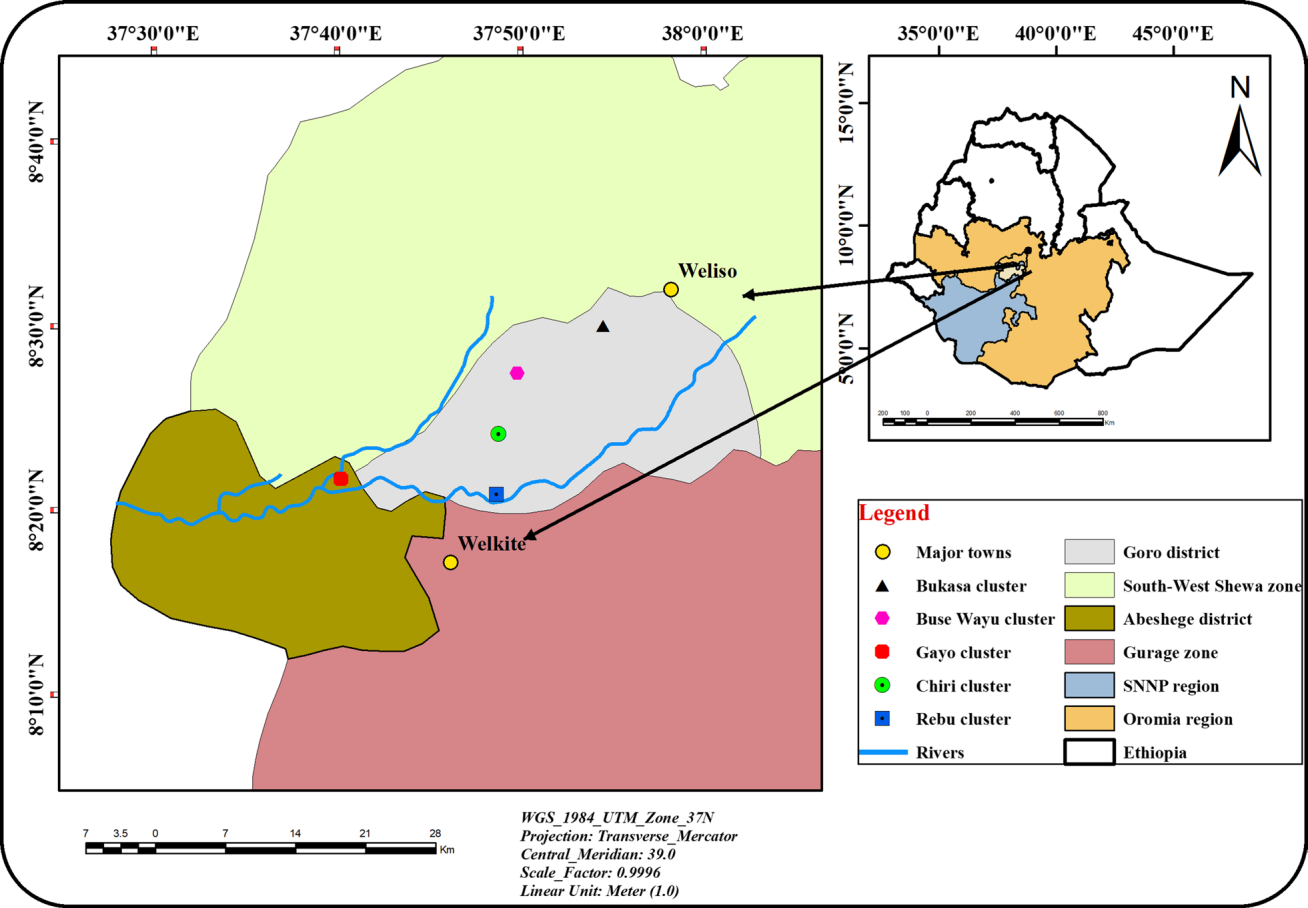
Map of study area

This study was conducted in two neighbouring kebeles, Rebu and Dire Lafto, which are located 144 km south west of Addis Ababa, the capital of Ethiopia (Fig. 1). Rebu kebele is located in the Goro district of the south west shewa Zone, Oromia Regional National State while Dire Lafto kebele is located in the Abeshege district, Southern Nations, Nationalities and Peoples Regional State, Ethiopia. Geographically located between latitudes 07^o^40′0′′N and 09^o^0′0′′N and longitudes 37^o^30′0′′E and 38^o^50′0′′E with mean altitude of 1748 m above sea level (Fig. 1).

Goro had a total population of 45,486, of which 22,912 were males and 22,574 were females according to the 2007 CSA report. From the total population, only 8.2% were urban dwellers. The majority (70.2%) of the inhabitants were Muslims, while 26.7% were Orthodox Christians and 2.2% were Protestants. Based on the 2007 Census conducted by the CSA, Abeshege Woreda had a total population of 61,424, of which 32,450 were males and 28,974 females; none of these were urban inhabitants. Half (50%) of the inhabitants were Orthodox Christians followed by Muslims (32.0%), Protestants (15.8%) and Catholics (1.3%) [23].

In the selected study sites, the major rainy season occurs from June to September, whereas the minor rainy season is from February to March. The sites are delineated by two intermittent streams, Rebu in the South East and Bala in the West, both of which have ample small water pools during the dry season.

### Mosquito collection

Adult mosquito collection was carried out monthly from November 2014 to July 2016. At the study sites, 5 clusters were chosen, with one in the vicinity of the Rebu River and one in the vicinity of the Bala River, whereas the other three clusters were at least 1.5–2.7 km away from any of the two rivers. In each cluster, 12 households were selected, making a total of 60 households. The average altitude for clusters Rebu, Gayo, Buse-Wayu, Chirri and Bukasa is 1769, 1614, 1813, 1766 and 1940 m above mean sea level, respectively. Four seasons were considered and the surveys represented wet season (June, July, August and September), transmission from wet to dry season (October and November), dry season (December, January, February and March) and transmission from dry to wet (April and May).

Indoor resting mosquitoes were sampled using Pyrethrum Spray Catches (PSCs) from the selected sixty houses from 06:00 am to 7:30 am, following a standard protocol [24] using insecticide aerosol (BAYGON^®^, S. C. Johnson & Son) procured from a local supermarket. Consent was obtained from the head of each household to conduct the monthly PSC and the head was informed one day prior to the PSC. Food and water were removed from the house and white sheets spread on the floor and over the furniture in the house. Two field workers, one inside the house and one outside, sprayed around the eaves. The mosquito collectors in the house then sprayed the roof and walls. The house was closed for 15 min after which the white sheets were brought outside to have sufficient light to see the dead and dying mosquitoes. After 15 min, dead and immobilized mosquitoes were collected and sorted by sex and genus. Collected mosquitoes were transported to a field laboratory for morphological identification.

### Mosquito processing

The mosquitoes were first classified into species using standard morphological keys [25] and then into different abdominal (gonotrophic) status, i.e. unfed, fed, half-gravid and gravid [26] at the field laboratory using a dissecting microscope. Then, each mosquito was kept in a labelled 1.5 ml Eppendorf tube containing silica gel desiccant and cotton wool. Samples were stored at − 20 °C at the Tropical and Infectious Diseases Research Center Laboratory, Jimma University until shipped to Imperial College London, VigiLab, UK for further processing. At the VigiLab, the mosquito samples were identified at species level using Polymerase Chain Reaction (PCR) following the protocols developed by Scott et al. for *Anopheles gambiae *sensu lato (*s.l*.) [27]. The presence of *Plasmodium* sporozoites and the source of the blood meal was assessed using PCR [28, 29].

### Statistical analysis

Mosquito abundance was analysed by a mixed Poisson regression model including household as random effect and season, distance to the river (close or away) and their interaction as categorical fixed effects. Human blood index (HBI) was calculated as the proportion of *Anopheles* samples with human blood (with possibly other species sources) out of the total number of *Anopheles* blood samples tested [30–32]. Bovine, goat and dog blood indices were calculated in the same way. The human blood index and sporozoite rate were compared between seasons using logistic regression model with household as random effect, and were restricted to mosquito catches from clusters close to the river as mosquito catches were too low for the three clusters away from the river.

The monthly entomological inoculation rate (EIR) of *Anopheles* mosquitoes was determined for each season as follows [33]:

$${\text{EIR}} = \frac{{{\text{no}}.\;{\text{ mosq}}\;{\text{fed }}}}{{{\text{no}}.\;{\text{human}}\;{\text{occupants}}}} \times \frac{{{\text{no}}.\;{\text{mosq}}\;{\text{fed}}\;{\text{on}}\;{\text{human}}}}{{{\text{no}}.{\text{ mosq tested for blood meal}}}} \times \frac{{\text{sporozoite rate}}}{{{\text{number of days}}/{\text{month}}}}.$$

## Results

### Seasonal density

A total of 2784 adult female *An. gambiae s.l.* were collected during the study period (Additional file 1). Of the 696 *An. gambiae s.l.* specimens analysed by PCR, all belonged to *Anopheles arabiensis*. The majority (99.1%) of the mosquitoes were collected from the clusters close to Rebu and Bala rivers. Table 1 shows the average daily household counts of *An. arabiensis* in the clusters close to and away from the river. The average daily household count was significantly higher (*P* = 0.037) in the clusters close to the river 5.35 (95% CI 2.41–11.85) compared to the clusters away from the river 0.033 (95% CI 0.02–0.05).

**Table 1:** The average daily household count (95% CI) of *Anopheles arabiensis* as a function of season and distance from the river

Season	Distance from the river	
	Close to the river	Away from the river
Wet	2.42 (0.83–7.08)	0.06 (0.02–0.12)
Transition wet–dry	14.56 (4.76–44.55)	0.04 (0.01–0.09)
Dry	4.53 (1.96–10.48)	0.01 (0.004–0.04)
Transition dry–wet	5.13 (2.15–12.21)	0.04 (0.02–0.10)
Total	5.35 (2.41–11.85)	0.033 (0.02–0.05)

Comparing the effect of vicinity of the river by season, a significant effect of closeness to the river was found during the dry season (*P* = 0.027) and transition from dry to wet season (*P* = 0.032), but not in the wet season (*P* = 0.084) and transition from wet to dry season (*P* = 0.090).

### Mosquito physiological state

**Fig. 2:**
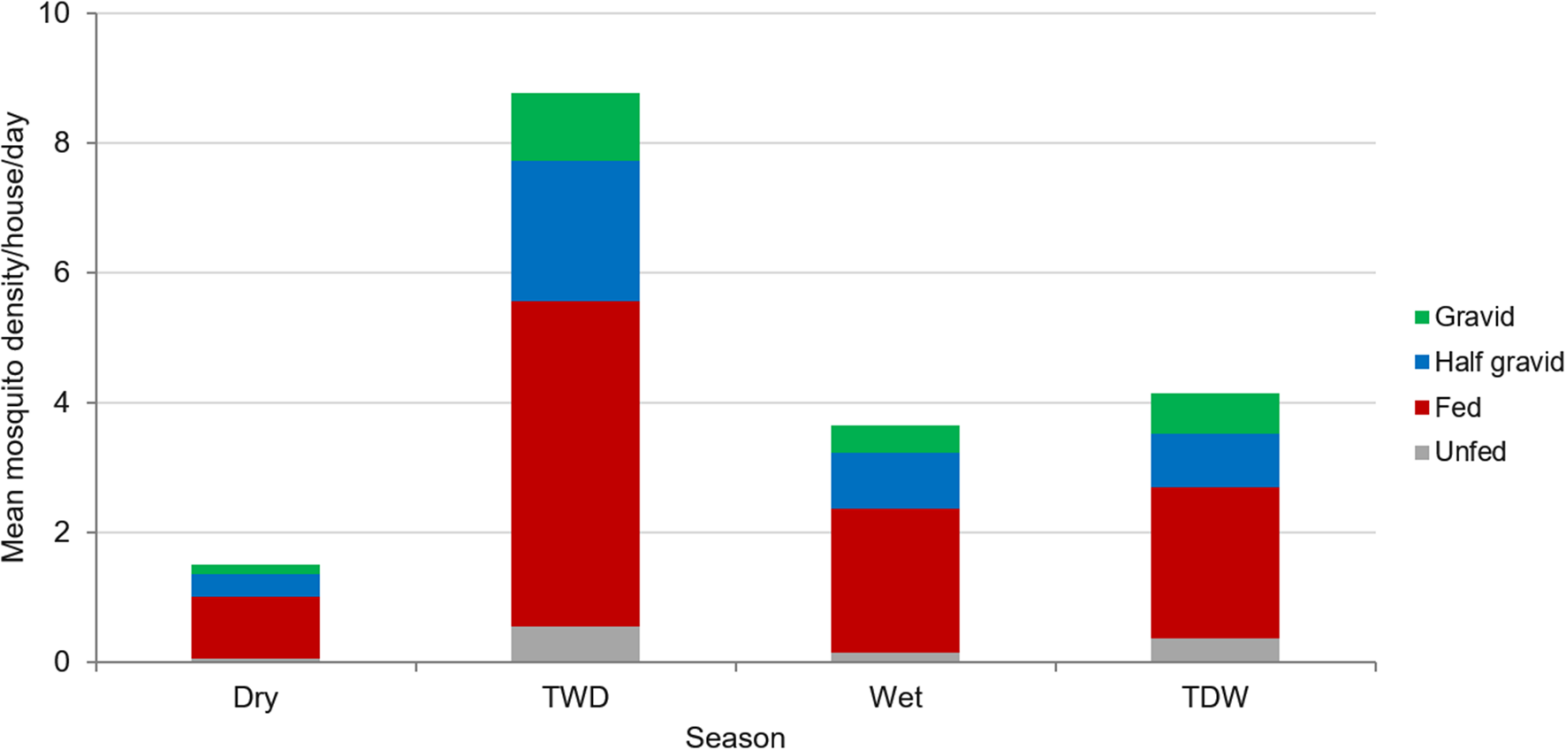
Physiological state of *Anopheles arabiensis*

The majority (58.9%) of the collected *An. arabiensis* were blood fed followed by half-gravid (23.5%), gravid (11.9%) and unfed (5.7%). The proportion of blood fed *An. arabiensis* was higher in all seasons (Fig. 2).

### Blood meal indices

Overall, *An. arabiensis* had higher bovine blood index (62.8%) as compared to human blood index (23.8%), ovine blood index (9.2%) and canine blood index (0.1%). The HBI of *An. arabiensis* during the wet, TWD, dry and TDW season was 23.3%, 14.5%, 26.6% and 28.2%, respectively (Table 2). There was no overall significant difference between the four seasons in terms of the HBI (*P* = 0.231).

**Table 2:** Number of blood samples (%) of *An. arabiensis* according to host species, season and vicinity to the river

Season	Close to the river								Away from the river			
	Human	Bovine	Goat	Dog	H + B	H + G	B + G	Unknown	Human	Bovine	H + B	Unknown
Wet	16 (14.8)	34 (31.5)	3 (2.8)	1 (0.9)	7 (6.5)	1 (0.9)	0	46 (42.6)	2 (25.0)	2 (25.0)	1 (12.5)	3 (37.5)
TWD	4 (2.6)	66 (43.4)	0	0	18 (11.9)	0	54 (35.5)	10 (6.6)	0	0	0	0
Dry	53 (14.4)	192 (52.0)	0	0	45 (12.2)	0	9 (2.4)	70 (19.0)	0	0	0	0
TDW	13 (12.0)	22 (20.4)	1 (0.9)	0	17 (15.8)	0	1 (0.9)	54 (50.0)	1 (50.0)	0	0	1 (50.0)
Total	86 (11.7)	314 (42.6)	4 (0.6)	1 (0.1)	87 (11.8)	1 (0.1)	64 (8.7)	180 (24.4)	3 (30.0)	2 (20.0)	1 (10.0)	4 (40.0)

*TWD* transition from wet to dry, *TDW* transition from dry to wet, *H + B* human and bovine, *H + G* human and goat; number in parentheses indicates percentage, *Unknown* unidentified blood meal sources

### Sporozoite and entomological inoculation rates

A total of 747 *An. arabiensis* samples were tested for *Plasmodium* circumsporozoite proteins (CSPs) using PCR (Table 3). Of these, 29 specimens were positive, 8 for *Plasmodium falciparum* and 21 for *Plasmodium vivax*. The overall *Plasmodium* sporozoite rate was 3.9% with 1.1% and 2.8% for *P. falciparum* and *P. vivax*, respectively. None of the mosquito specimens collected from clusters away from the river were positive for CSP (Table 3).

**Table 3:** Sporozoite rates of *An. arabiensis* in clusters close to and away from the river according to season

Season	Close to the river			Away from the river		
	No tested	Pf + ve (%)	Pv + ve (%)	No tested	Pf + ve (%)	Pv + ve (%)
Wet	108	2 (1.85)	4 (3.70)	8	0	0
TWD	152	2 (1.32)	2 (1.32)	0	0	0
Dry	369	4 (1.08)	12 (3.25)	0	0	0
TDW	108	0 (0.00)	3 (2.78)	2	0	0
Total	737	8 (1.09)	21(2.85)	10	0	0

*TWD* transition from wet to dry, *TDW* transition from dry to wet, *Pf + ve* positive for *P. falciparum* circumsporozoite protein (CSP), *Pv + ve* positive for *P. vixax* CSP, *%* percentage

The entomological inoculation rate was determined only for the clusters close to the river due to low mosquito count for clusters away from the river. The overall *P*. *falciparum* and *P. vivax* EIR for *An. arabiensis* was 0.18 and 0.07 infective bites per person/month, respectively. Peak *P. falciparum* EIR was observed during the TWD season with EIR of 0.13 infective bites per person/month whereas peak *P. vivax* EIR was observed during the TDW and dry season with EIR of 0.18 infective bites per person/month in those seasons.

## Discussion

Understanding the seasonal dynamics of malaria-vector populations and malaria transmission intensity in semi-arid areas, where breeding sites are limited to rivers during dry season, is essential to design effective strategies for malaria control and elimination. Moreover, accurate information on dynamics of malaria transmission and its spatial distribution helps to define when and where the largest infection risks occur, and facilitates the development of appropriate and targeted control strategies [17]. In the present study, key entomological parameters such as species composition, seasonal distribution, physiological status, blood meal sources, sporozoite rates and EIR were assessed in a semi-arid area to determine seasonal dynamics of mosquitoes and transmission intensity in clusters close and away from intermittent rivers. The EIR was used as a surrogate for malaria transmission intensity, as no data on the incidence of malaria cases were available. Thus, understanding the heterogeneity in malaria vector density and transmission intensity along rivers enables spatially targeted mosquito vector control.

In this study, all specimens of *An. gambiae* *s.l.* identified by the PCR were *An. arabiensis*. In line with the study, the presence of a single malaria vector species in a locality has also been observed elsewhere [19, 34, 35]. *Anopheles arabiensis* is certainly the dominant species in south west Ethiopia [36], whether using the PSC, the CDC light trap, exit traps or the artificial pit shelter. In this study, only PSC was used as a method of mosquito collection, which might have been the reason why we only observed *An. arabiensis*. Other mosquito species, such as *Anopheles coustani* and *Anopheles pharoensis* might be present in the study region, but as they are exophilic they are rarely captured by indoor collection methods like PSC. *Anopheles arabiensis* was the predominant anopheline species in lowland areas of Kenya [37], south-central Ethiopia [38] and south-west Ethiopia [39, 40]. This might be due to the fact that *An. arabiensis* adopts better to hot and arid climatic conditions [41].

Previous studies documented higher *Anopheles* mosquito abundance in houses near the river than houses far from the river [42–44]. Similarly, in this study, the mean density of the *An. arabiensis* was significantly higher in clusters close to the river compared to clusters away from the river. This could be explained by the availability of breeding sites in riverbeds [19, 45–48].

A significant effect of proximity to the river was found during the dry season and transition from dry to wet season, whereas no statistically significant effect of closeness to the river was found in the wet and transition from wet to dry seasons. This might be due to the fact that riverbeds were the only breeding sites in the study region during dry and transition from dry to wet seasons whereas there were ample breeding sites in all clusters during the wet and transmission from wet to dry seasons. The findings of this study match with the results of the previous studies [35, 49] which recorded variation in vector densities over seasons. The results also showed that the bovine blood index (BBI) of *An. arabiensis* was higher compared to the human blood index (HBI) and this is in line with previous reports [50–52]. The unidentified blood meal sources could be of donkey, horse, sheep and chicken for which we did not test. The zoophilic behaviour of *An. arabiensis* observed in the present study is consistent with the results of other studies [39, 53–57].

The overall sporozoite rate was 3.9% and 0% for clusters close to and away from the river, respectively. This is comparable with earlier reports in Africa where sporozoite rates in *An. arabiensis* ranged from 2 to 7% [58, 59]. In southern and south-central Ethiopia, much lower sporozoite rate of *An. arabiensis* for *P. falciparum* was documented [38, 39, 60]. The variation in sporozoite rate could be related to the different feeding behaviour of *An. arabiansis*. Sporozoite rates are typically lower when the vector is more zoophilic [61] which is consistent with the findings of this study. Sporozoites were only found in clusters close to the river. Similarly, a clustered distribution of *Plasmodium* CSPs-positive *An. arabiensis* in a sub-village near the shore of a lake was observed in Ethiopia [20, 39]. This study is in line with a study conducted in Eastern Sudan where CSP was higher in villages along Rahad river basin [19].

The overall *P. falciparum* and *P. vivax* EIR of *An. arabiensis* in clusters close to the rivers in Goro and Abeshege districts were 0.8 and 2.19 infective bites per person/year, respectively. This indicates that the entomological inoculation rates in the study area were in agreement with previous reports [42, 62]. The results are also consistent with the findings in Tanzania [4] where malaria transmission was strongly associated with proximity to the river. The EIR in the present study is substantially higher than the one found in southern Ethiopia where *P. falciparum* and *P. vivax* EIR of *An. arabiensis* was 0.1 infective bites per person/year for houses close to a breeding site [52]. *Plasmodium* CSP positive *An. arabiensis* were abundant in clusters close to river, similar to the findings of previous studies [20, 39, 42]. High *P. falciparum* EIR was recorded during transition from wet to dry season whereas high *P. vivax* EIR was recorded during transition from dry to wet season. This indicates that malaria transmission persists immediately after the wet season and late in the dry season [14].

## Conclusions

This study showed that riverbeds contribute substantially to malaria transmission during the dry and transition from dry to wet seasons suggesting that riverine breeding sites are the main driving transmission hotspots in the area. Infectivity and transmission intensity were recorded in clusters close to the river when the rivers became intermittent and large number of pockets of breeding sites were created. The results presented here support that vector control strategies to eradicate mosquitoes should target the larval stage at the time when the mosquito population is at its lowest and confined to few remaining hotspots in the semi-arid areas of central Ethiopia. Moreover, identifying and assessing mosquito breeding sites using Geographic Information System (GIS) is useful with river locations as a tool for more effective larval source management in the highlands of Ethiopia. Since clustering of malaria transmission intensity was observed, targeted vector control interventions should be implemented to reduce the overall transmission and sustain malaria control to move towards elimination.

## Supplementary Information

**Additional file 1** Mosquito counts.

## Data Availability

Data are available from the corresponding author upon request.

## Abbreviations

EIR: Entomological inoculation rate
TWD: Transmission from wet to dry
TDW: Transmission from dry to wet
Pf + ve: Positive for *P. falciparum* circumsporozoite protein (CSP)
Pv + ve: Positive for *P. vixax* circumsporozoite protein
%: Percentage
H + B: Human and bovine
H + G: Human and goat
PSC: Pyrethrum space spray catches
CSP: Circumsporozoite protein
